# Compressive deformability and flexural fracture energy of high-strength concrete with bamboo fibers

**DOI:** 10.1038/s41598-026-48624-x

**Published:** 2026-05-05

**Authors:** Maciej Kaźmierowski, Maciej Siemiginowski, Michał Kordasz, Michał Drzazga

**Affiliations:** 1 Department of Civil Engineering, Faculty of Environmental Engineering and Geodesy, Wrocław University of Environmental and Life Sciences, 50-365 Wrocław, Poland; 2 Department of Graphics, Computer Vision and Digital Systems, Faculty of Automatic Control, Electronics and Computer Science, Silesian University of Technology, 44-100 Gliwice, Poland

**Keywords:** Engineering, Materials science

## Abstract

Knowledge of the behavior of high-strength concrete reinforced with bamboo fibers remains limited with respect to compressive deformability and flexural fracture energy. The effect of alkali-treated bamboo fibers (1.0% and 1.5% by weight of cement; 2% NaOH) on the fresh-mix properties, mechanical performance, compressive deformability, and flexural fracture energy of high-strength concrete was evaluated. The addition of fibers increased the air content and reduced the consistency of the mix. The compressive strength changed by + 4% and − 6%, while the strain corresponding to peak stress increased by 11% and 7%. The splitting tensile strength decreased by 14%, whereas the flexural tensile strength increased significantly by 12%. A more pronounced effect was observed for fracture energy, which increased significantly: G_f,δ_ by 20% and 143%, and G_f, CMOD_ by 20% and 33%. The increase in fracture energy may be associated with delayed microcrack initiation in the notch-tip zone, limited microcrack coalescence, and short-term load retention near the peak value, as confirmed by the flexural response curves and by the analysis of the evolution of the principal tensile strain ε₁ concentration zone. Scanning electron microscopy revealed only minor qualitative changes in fiber surface morphology after alkali treatment.

## Introduction

One approach to improving the mechanical performance of concrete and limiting brittle fracture is the use of dispersed reinforcement in the form of fibers, including bamboo fibers^1^. Bamboo is biodegradable^2^, widely available, fast-growing^3^, and can be industrially processed at relatively low cost^4–6^; moreover, it exhibits a favorable strength-to-weight ratio^7^. The physical and mechanical properties of bamboo fibers, including density, tensile strength, and elastic modulus, vary depending on the species (more than 1200 species have been identified^1^, as well as on growth conditions, moisture content, and the methods of extraction and treatment^8^. The ranges typically reported in the literature are as follows: density of 500–900 kg/m^3^, tensile strength of 130–400 MPa, and elastic modulus of 10–30 GPa^9–11^. Numerous studies have confirmed the suitability of bamboo as a reinforcing material in cementitious composites^12–14^.

However, the literature also indicates significant limitations associated with the use of bamboo as reinforcement in cementitious composites^15^. The main drawbacks include high hygroscopicity^11^, limited chemical resistance in alkaline environments^4,16^, and the presence of organic constituents, including hemicellulose and lignin, which reduce adhesion to the cement matrix^7,17^. Limited fiber ductility is another important issue^15,18^. Studies have shown that bamboo fiber modification, including hot-water treatment^8,19^, exposure to alkaline solutions^2,20^, microwave irradiation^21^, and the application of polymer coatings and natural resins^22^, may improve durability, chemical resistance in alkaline environments, and matrix adhesion, which is associated with enhanced chemical bonding and increased friction at the fiber–matrix interface^4,7,14,21,20,23^. It should be noted, however, that excessively high alkali concentrations—the least expensive and most commonly used method of chemical fiber treatment^24^—may lead to excessive surface degradation, including the removal of cellulose and lignin, and consequently to reductions in fiber strength and stiffness^18,25,26^.

The concentration of the alkaline solution and the exposure time are critical for removing undesirable fiber constituents, such as the waxy layer, hemicellulose, and part of the lignin, while preserving the structural integrity of the fiber^24^. The literature has also reported improvements in the mechanical performance of cementitious composites incorporating bamboo fibers without chemical treatment^4,6,11,27,28^. This indicates that the absence of alkali treatment, although not ensuring optimal adhesion, does not preclude a reinforcing effect, including crack-bridging action within the composite.

In recent years, numerous studies have been published on cementitious composites modified with bamboo fibers, including conventional concrete and other cement-based composites^6,21,27,29–31^, self-compacting concrete^2,32^, and cement mortars^33,34^. These studies differed not only in matrix type, but also in fiber length, fiber content, and fiber preparation method, including the use of untreated fibers, alkali-treated fibers, or fibers modified by other methods^2,21,32,33,30,34^. As a result, the effect of fibers on mechanical properties has been inconsistent. Some studies reported improvements in compressive strength^2,6,29,32,33^, splitting tensile strength^2,6,21,32^, or flexural strength^6,21,27,29,32,33^, whereas others found the influence of fibers on selected mechanical properties to be limited or even unfavorable^6,30,31,34^. A similar variability of results has also been highlighted in a review paper^1^.

Bamboo fibers have been reported to effectively limit crack propagation and increase the fracture-mechanics parameters of composites; however, data on the fracture energy of high-strength concrete reinforced with bamboo fibers remain limited. In lightweight aggregate concrete, the greatest improvement was obtained for fibers 30 mm in length and a volumetric dosage of 0.75%, for which an approximately threefold increase in fracture energy was reported^31^. In cement-paste-based composites, a 45-fold increase in fracture resistance was observed after the incorporation of thin bamboo cellulose fibers with a length of 2.5 mm, a diameter of approximately 13 μm, and a dosage of 16% by mass^35^. In normal-strength concrete, it was demonstrated that the length and content of raw bamboo fibers, as well as aggregate size, affect fracture-mechanics parameters, and that the presence of fibers promotes the extension of the microcrack propagation path and an increase in fracture energy, which, under the optimal configuration, reached approximately 90% for 1.5% fiber volume, a fiber length of 20 mm, and a maximum aggregate size of 10 mm^4^. In mortars modified with coal fly ash and bamboo fibers derived from composite-manufacturing waste, fibers with greater slenderness were found to be more effective in limiting crack propagation^34^. These findings indicate that the effectiveness of bamboo fibers in shaping fracture-mechanics parameters depends on their geometry, dosage level, and the properties of the cementitious matrix. Consequently, further in-depth studies on high-strength concrete modified with bamboo fibers remain justified^1^.

The fracture-mechanics parameters of concrete may be governed not only by the presence of fibers, but also by the binder composition and the type of aggregate. It has been shown that properly designed multicomponent cements containing fly ash, silica fume, and nanosilica may lead to improved fracture-mechanics parameters relative to composites based solely on Portland cement, whereas unfavorable proportions of mineral additions may produce the opposite effect^36,37^. It has also been demonstrated that the type of coarse aggregate significantly affects fracture-mechanics parameters, with more favorable values reported for concretes made with limestone, granite, and basalt aggregates than for concretes containing gravel aggregate^38^. A different effect has been observed in composites incorporating recycled aggregates and recycled powders, in which reduced fracture-mechanics parameters and greater damage development intensity were reported. Acoustic emission studies linked this phenomenon to the presence of old mortar and weakened interfacial transition zones between aggregate particles and the matrix^39^.

It should be emphasized that studies on high-strength concrete reinforced with bamboo fibers remain scarce, although differences in cement-matrix microstructure between high-strength and normal-strength concretes may significantly affect the load-transfer mechanism^20,40,41^. The mechanical efficiency of fibers depends to a large extent on the structural properties of the cementitious matrix, as confirmed, among others, for normal-strength concretes in which the addition of silica fume—a typical constituent of high-strength concretes—increased matrix compactness and promoted improved adhesion^42^. Due to their hygroscopic nature, plant fibers tend to swell, which may lead to degradation of the fiber–matrix interfacial zone^33^. A low water-to-binder ratio (w/b), which reduces the content of free water in the matrix, may help to limit this phenomenon. This feature is characteristic of high-strength concrete and provides an additional rationale for conducting research in this field^11^.

It should also be noted that, to date, no analyses of high-strength concrete reinforced with bamboo fibers subjected to typical alkali treatment^1,2^ have been reported using digital image correlation (DIC), either in compression tests or in three-point bending tests on notched beams. DIC makes it possible to quantitatively describe compressive deformability both in a linear sense, including σ–ε curves obtained from a virtual extensometer, and in an area-based sense, while in bending it enables evaluation of the evolution of the principal tensile strain ε₁ concentration zone at the notch tip.

The aim of this study was to quantitatively evaluate the effect of bamboo fibers on the fresh-mix properties and the material characteristics of high-strength concrete, with particular emphasis on compressive deformability and flexural fracture energy in notched beams. The results were subjected to statistical significance analysis, and DIC measurements were used to analyze deformations in compression in both linear and area-based terms, as well as to assess the evolution of the principal tensile strain ε₁ concentration zone at the notch tip. The experimental program was complemented by microstructural analysis and a modified digital procedure for fiber length assessment. The obtained results supplement the limited body of data on high-strength concrete reinforced with bamboo fibers and may serve as a reference point for further studies on these composites.

## Materials and methods

### Experimental program

The experimental program comprised three series of concrete mixtures with different contents of bamboo fibers, dosed as a percentage of cement mass: Series A—0%, Series B—1.0%, and Series C—1.5%. The program included the determination of fresh-mix properties, namely consistency and air content, as well as the mechanical properties of hardened concrete in compression, splitting tensile, and three-point bending tests on notched beams, including fracture energy determined from the δ and CMOD curves. The compression and bending tests were complemented by digital image correlation (DIC) analysis. The program was further extended to include fiber length assessment and microstructural observations of the composite and the fibers before and after alkali treatment. The adopted experimental program was multi-stage in nature and comprised complementary stages of material evaluation, enabling an integrated interpretation of the mechanical results, DIC analysis, and microstructural observations^43^.

### Materials and mixture composition

**Fig. 1:**
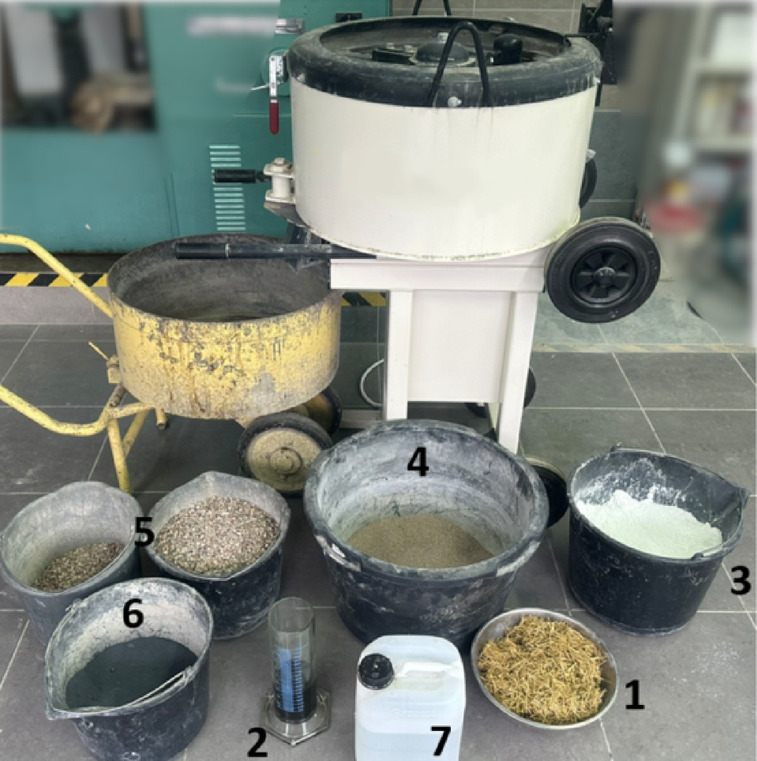
Constituents of one batch of the concrete mixture for Series B and the preparation setup: (1) bamboo fibers; (2) superplasticizer; (3) cement; (4) sand; (5) gravel aggregate; (6) silica fume; (7) water.

The concrete mixtures were prepared using the following constituents: white Portland cement CEM I 52.5 R, with a specific surface area of 4210 cm^2^/g (determined by the Blaine method using a manual apparatus^44^ and a specific density of ρ = 3050 kg/m^3^; natural fine aggregate in the form of washed sand with a particle size range of 0–2 mm (ρ = 2550 kg/m^3^); gravel aggregate with a fraction of 2–8 mm (ρ = 2660 kg/m^3^); a high-range water-reducing admixture in the form of a polycarboxylate-based superplasticizer (ρ = 1120 kg/m^3^); and silica fume with an SiO_2_ content of ≥ 95%, a dominant particle fraction of < 100 μm, and a density of ρ ≈ 2300 kg/m^3^. The water-to-binder ratio (w/b) of the mixture was 0.34. The mixture compositions for the individual series are summarized in Table 1. The fiber dosage levels were selected based on previous literature reports^1,45^. Figure 1 shows the constituents used to prepare one batch of the mixture for Series B.

**Table 1:** Concrete mixture composition for the individual series.

Components	Unit	Series		
		A	B	C
Bamboo fibers	kg/m^3^	0	5.96	8.94
	%^*^	0	1.0	1.5
Superplasticizer	l/m^3^	25.5		
Cement (CEM I 52.5 R)	kg/m^3^	596		
Sand (0–2 mm)	kg/m^3^	500		
Gravel aggregate (2–8 mm)	kg/m^3^	990		
Silica fume	kg/m^3^	59.6		
Water-to-binder ratio (w/b)	[-]	0.34		

^*^ percentage by mass of cement.

The 0–8 mm aggregate was characterized by continuous grading, without distinct gap fractions. The proportion by mass passing the 4 mm sieve was 75.6%, while that passing the 2 mm sieve was 48.4% (with the 2–4 mm fraction accounting for 27.2%); the content of particles smaller than 0.063 mm was 1.8%. This grading distribution is consistent with the typical aggregate gradation used in concretes with a reduced w/b ratio, and the proportion of the < 0.063 mm fraction does not indicate an elevated fines content^46^.

### Bamboo fiber preparation and characterization

**Fig. 2:**
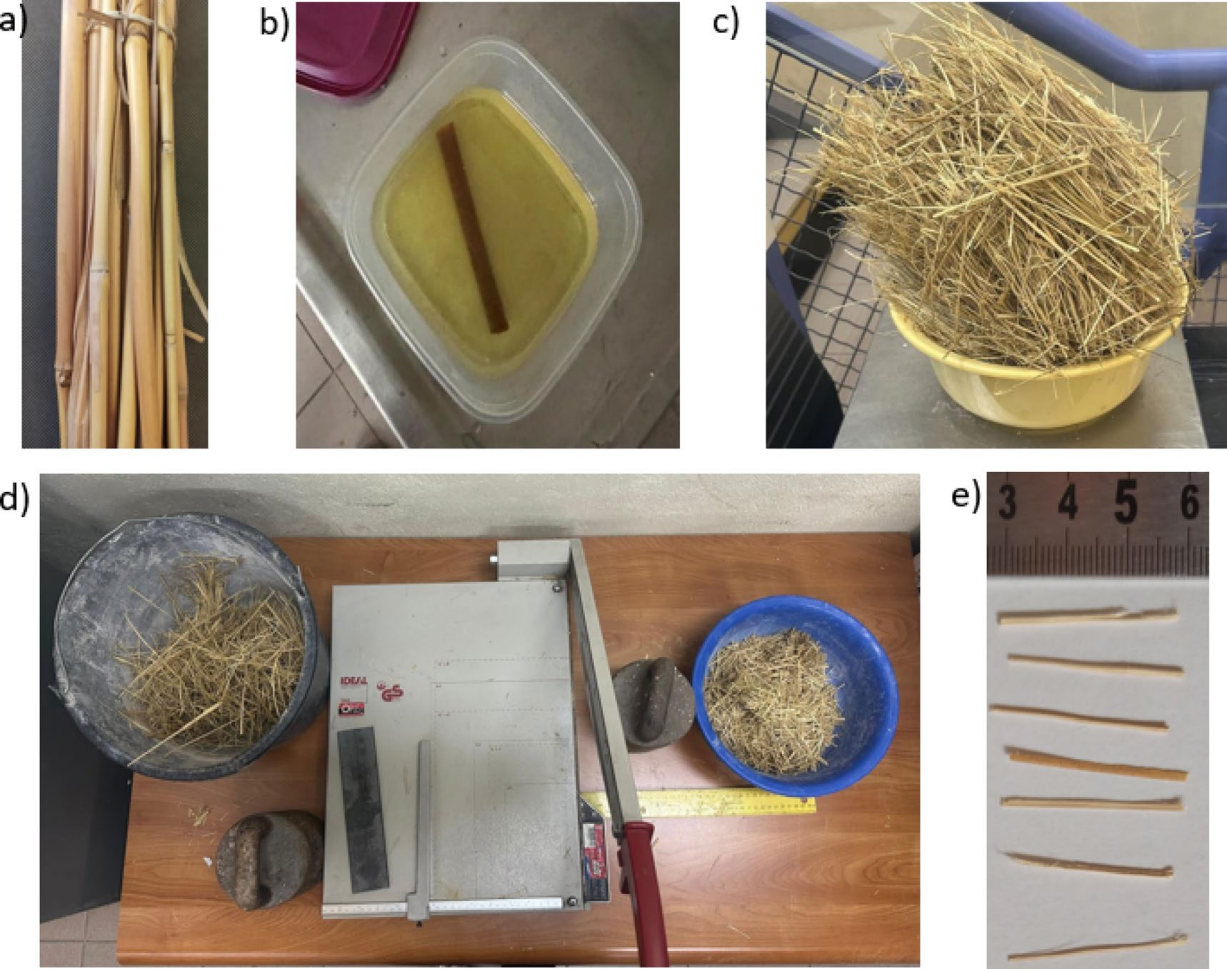
Procedure for preparing bamboo fibers for the experimental program (described in the text).

Phyllostachys reticulata (Tonkin bamboo) fibers were used in the study (Fig. 2a). The bamboo poles used for fiber preparation were purchased in Poland as a commercial product. Due to the time elapsed, detailed information on the supplier and batch identification was no longer available. The material was not collected from the natural environment; therefore, no harvesting permits were required. The preparation procedure was developed based on the method described in Ref.^1^, with modifications to the drying temperature and the inclusion of an additional rinsing step. In the first stage, the bamboo poles were cut into sections approximately 120 mm in length and then soaked in water for 24 h. After soaking, the material structure was mechanically separated by gentle hammering followed by manual defibration. The fibers were then subjected to alkali treatment by immersion in a 2% sodium hydroxide solution (NaOH, purity >99%) for 24 h (Fig. 2b). The solution concentration and exposure time were selected in accordance with literature data^1,2,45^. After treatment, the fibers were thoroughly rinsed with water to remove residual alkali, thereby reducing the risk of excessive fiber degradation^21^. The fibers were then dried at 40 °C until constant mass was achieved to ensure repeatable dosing conditions in the dry state. In the final stage, the fibers were cut using a manual guillotine into segments approximately 30 mm in length (Fig. 2d–e). A view of the prepared fibers intended for incorporation into the concrete mixture is shown in Fig. 2c.

**Fig. 3:**
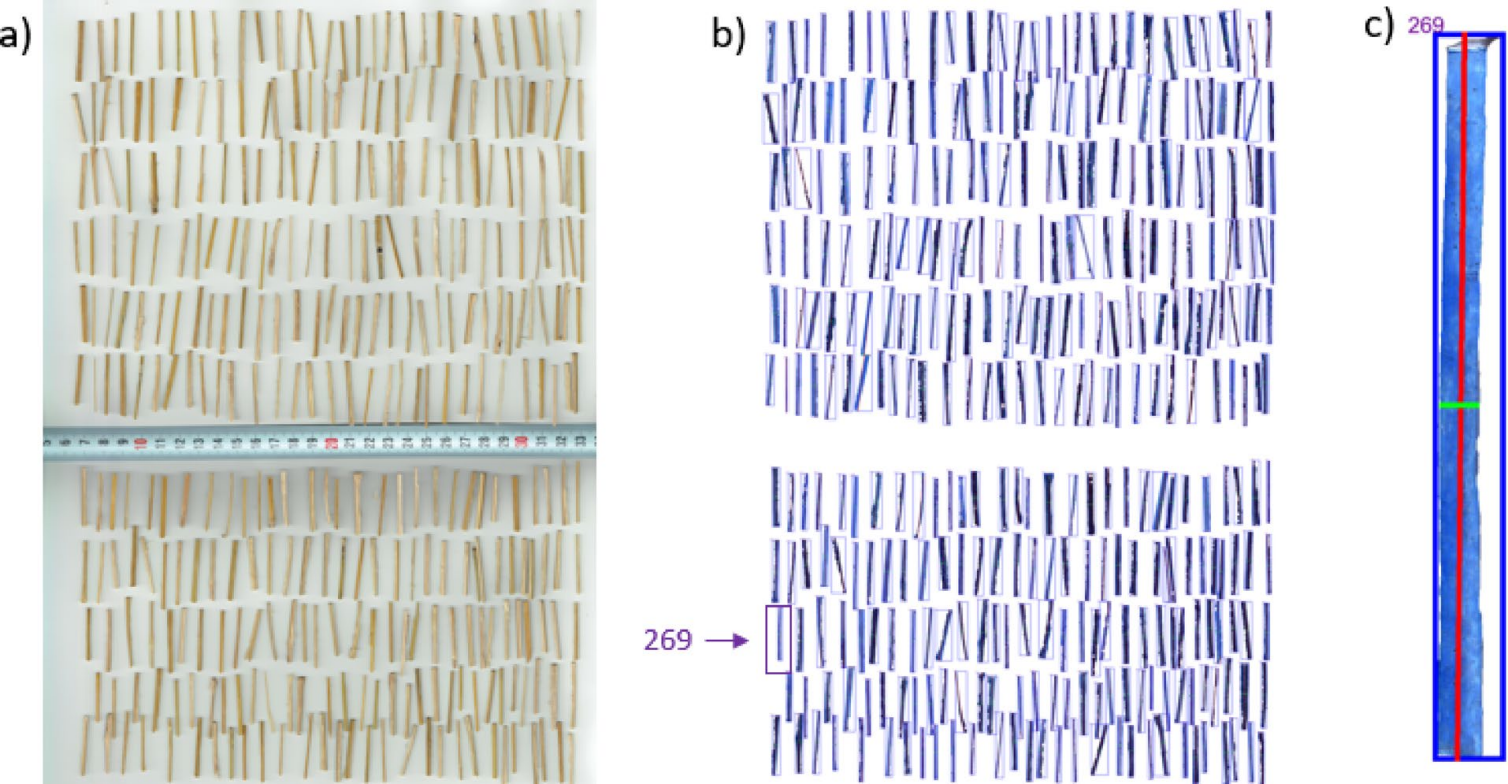
Determination of bamboo fiber length by image analysis: (**a**) scan with the calibration scale; (**b**) fibers extracted from the image (segmentation result); (**c**) enlarged view of fiber No. 269 extracted during segmentation, with the longitudinal and transverse axes marked.

Fiber length was verified by means of image analysis. A total of 342 fibers were randomly selected and placed on the flat surface of a scanner without mutual contact, together with a visible linear scale for calibration (Fig. 3a). Scanning was performed at an optical resolution of 2400 DPI using a Brother MFC-J6520DW scanner. The files were saved in lossless TIF format without additional image processing. The analysis was carried out in the Python environment (v3.11) using the scikit-image library (v0.26), which enabled object segmentation and labeling as well as the determination of object dimensions in pixels.

**Fig. 4:**
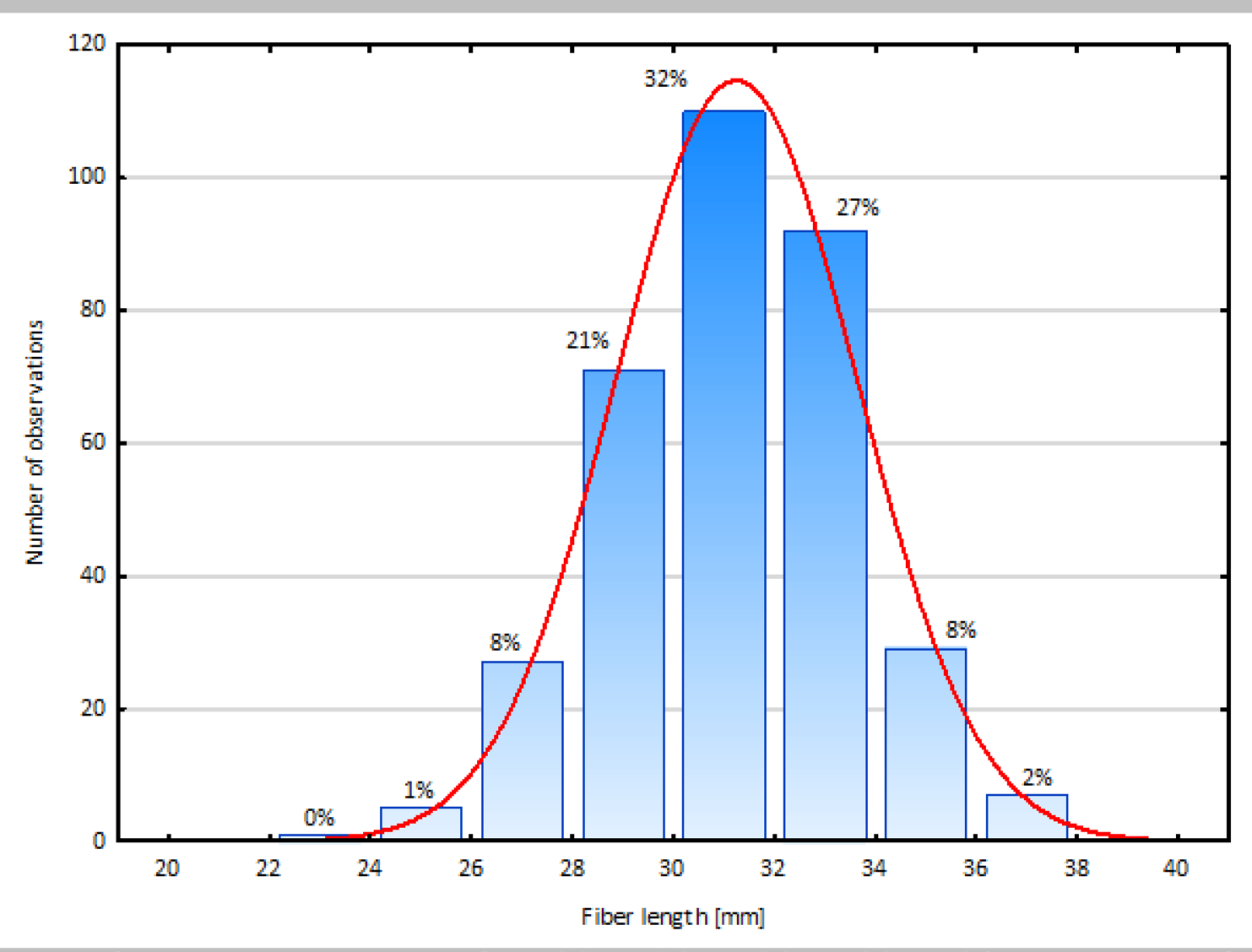
Histogram of bamboo fiber length distribution with the fitted normal distribution curve (red line).

The length of an individual fiber was defined as the Euclidean distance between the two extreme points of the object determined along its longitudinal axis (Fig. 3c). This value was then converted into real units based on calibration against the scale placed in the same scan (Fig. 3a), where 1 mm corresponded to 50 px. The px/mm value was determined directly from the linear scale and adopted as the basis for conversion independently of the nominal DPI value. Under this calibration, the conversion resolution was 0.02 mm/px. Incorrectly segmented objects, such as fibers in contact, partially overlapping fibers, fibers truncated at the scan edge, or markedly curved fibers, were rejected during the quality-control stage. An example segmentation result is shown in Fig. 3b–c. The resulting dataset was subjected to descriptive statistical analysis and assessment of distribution normality using Statistica software (Fig. 4).

Due to the irregular cross-sectional shape of the fibers, their width was not determined by image analysis. Instead, the width was measured with a caliper (0.01 mm resolution) on a sample of *n* = 30 fibers, yielding a mean value of 0.39 mm.

### Mix procedure and specimen preparation

The constituents of the concrete mixture were proportioned by weight with an accuracy of 1%. The sequence of batching and mixing was as follows: first, the coarse aggregate was mixed with the sand, followed by the addition of cement and silica fume. After dry mixing of these constituents, water containing the superplasticizer was introduced. The bamboo fibers were added as the final constituent. The total mixing time was approximately 8 min. The specimens for testing were cast, stored, and cured in accordance with the standard^47^.

### Test methods

The consistency of the concrete mixture was determined using the slump test^48^(F_T_) and the Vebe test^49^(V_B_). The air content of the mixture (A_air_) was determined using a pressure meter in accordance with the standard^50^. Each determination was performed in three replicates for each series. The hardened concrete properties were tested 28 days after casting. Before the compressive strength and splitting tensile strength tests, the density of the specimens was determined by the hydrostatic method. The specimens were then positioned axially in the loading system (centered) and subjected to a preload.

**Fig. 5:**
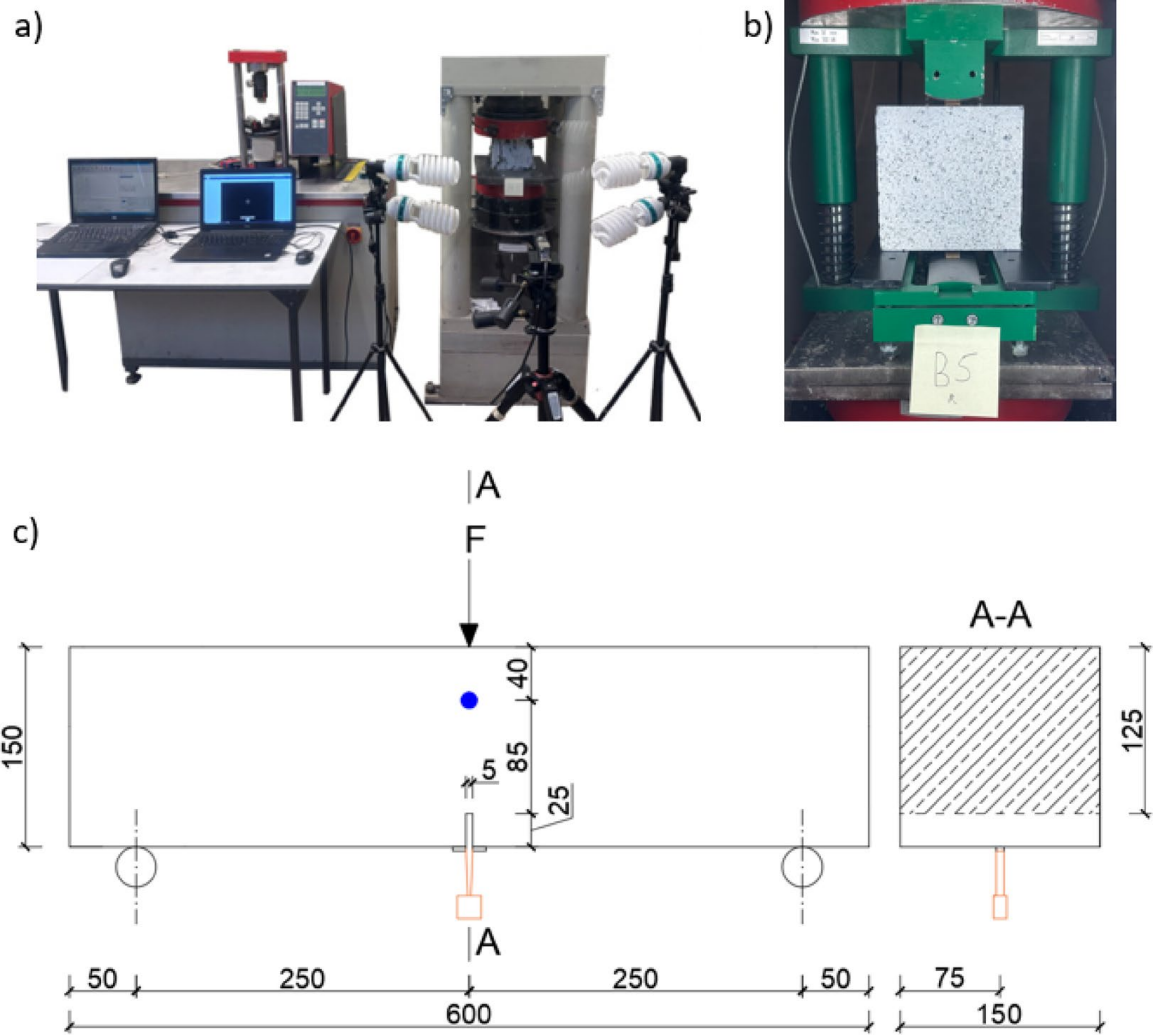
Test setups for determining: (**a**) compressive strength; (**b**) splitting tensile strength; (**c**) flexural tensile strength ( deflection measurement point δ determined by DIC; ― clip gauge extensometer measuring crack mouth opening displacement, CMOD).

The compressive strength (f_c_) was determined in accordance with the standard^51^ using a hydraulic testing machine with a maximum load capacity of 3000 kN (Fig. 5a) and cube specimens with a side length of 150 mm. Loading was applied under piston displacement control at a rate of 0.07 mm/min until specimen failure. This value was adopted on the basis of Ref. ^52^ and in order to record the stress–strain curve also in the post-peak range.

The splitting tensile strength (f_ct, sp_) test was performed in accordance with the standard^53^ on cube specimens with a side length of 150 mm (Fig. 5b). The load was applied under piston displacement control at a rate of 0.4 mm/min using the same testing machine as in the compression test.

The flexural tensile strength (f_ct, fl._) was determined based on the assumptions of the standard^54^ by recording the beam deflection (δ) and the crack mouth opening displacement (CMOD). On this basis, the fracture energy of the composite determined from the δ and CMOD curves was calculated (G_f,δ_​ and G_f, CMOD_, respectively). The beams were loaded in a three-point bending configuration under piston displacement control at a rate of 0.2 mm/min using a testing machine with a maximum load capacity of 100 kN. CMOD was measured using a clip gauge extensometer (Fig. 5c).

### DIC procedure and data processing

In the compressive strength test, deformations on the lateral surface of the specimen were recorded using digital image correlation (DIC) with the Aramis system (GOM/ZEISS, Germany). The strain in the loading direction was determined using a virtual extensometer with a gauge length of 80 mm, positioned centrally on the observed surface and oriented along the compression axis. The camera, placed at a distance of approximately 30 cm from the specimen, recorded images at a resolution of 4096 × 3000 pixels and a frequency of 1 Hz. The DIC analysis parameters were as follows: facet size of 15 × 15 pixels and facet point spacing of 7 pixels.

Beam deflection (δ) was recorded using the DIC method. The deflection measurement point was defined at a distance of 85 mm from the notch tip in the direction of the beam height (Fig. 5c). The acquisition frequency for load, CMOD, and δ was 2 Hz. The camera-to-specimen distance was also approximately 30 cm.

**Fig. 6:**
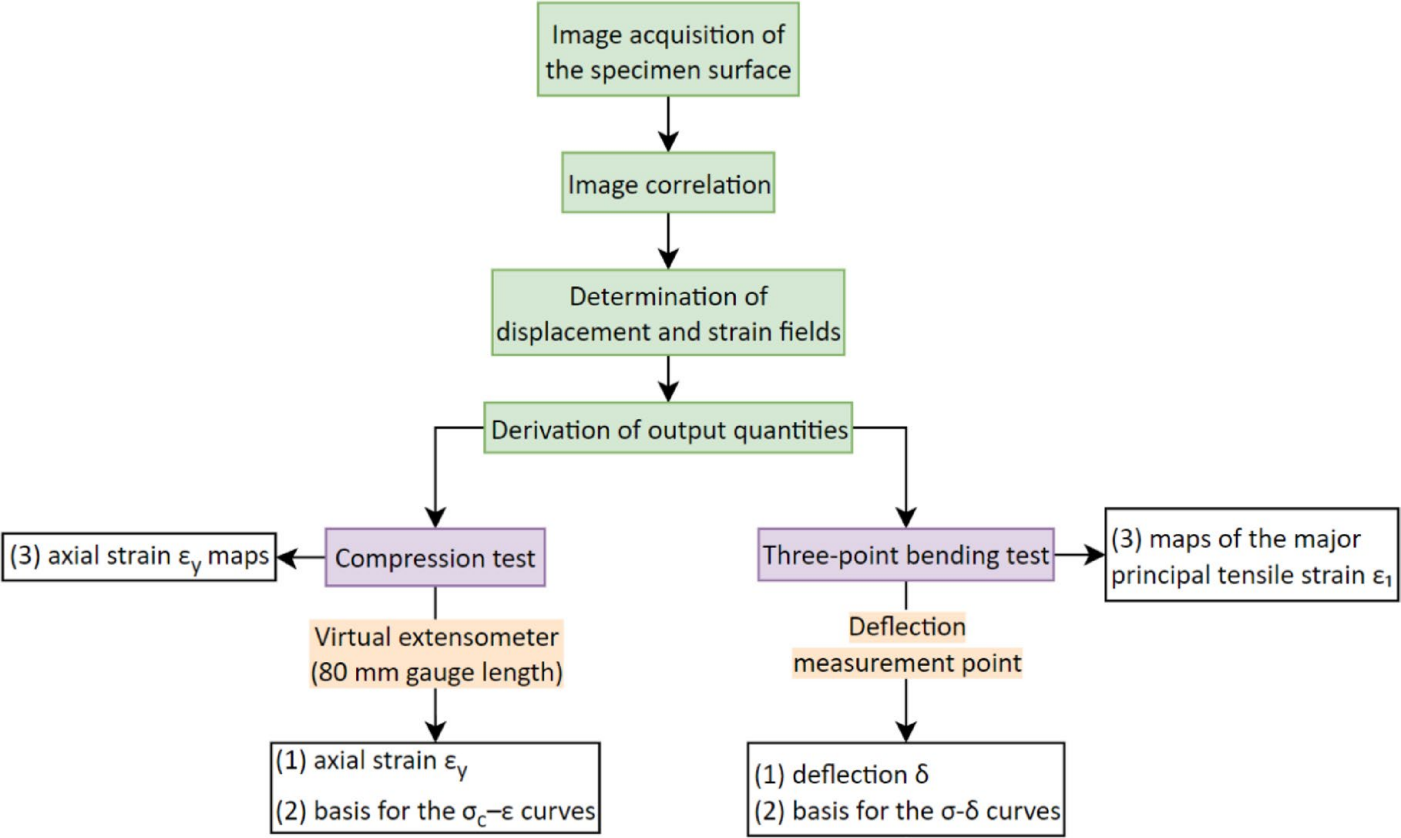
Flowchart of the DIC procedure applied in this study, including image acquisition, image correlation, determination of displacement and strain fields, and derivation of the quantities used in the compression and three-point bending analyses.

Before DIC acquisition, the specimen surfaces were prepared by applying a random speckle pattern consisting of a white background with black speckles, while uniform illumination was provided by LED lamps positioned to reduce glare and shadows. The images were recorded using the Aramis Adjustable 2D system and then analyzed in the Aramis SRX environment used for further processing of the recorded image series. In the applied procedure, no separate calibration plate was used. The image scale was established based on a reference scale placed in the reference image by defining a reference segment of known actual length. The main stages of the DIC procedure applied in this study, together with the quantities used in the subsequent analysis, are shown in Fig. 6.

### Microstructural observations

Observations of the fracture surface of the specimen after compression were performed using a VHX-7000 N digital microscope (Keyence, Japan) in the fiber–matrix interfacial region. Point elemental analysis was performed in a selected microregion using an EA-300 head (Keyence) and laser-induced breakdown spectroscopy (LIBS). The LIBS results were treated as a qualitative identification of the composition within the micro-area. The surface morphology of the fibers in the raw state and after alkali treatment (fibers not incorporated into the concrete mixture) was assessed by scanning electron microscopy (SEM) using an EVO^®^ LS 15 microscope (ZEISS, Germany) equipped with a secondary electron detector (SE1), at an accelerating voltage of 20 kV and a magnification of 300×. Samples taken from the inner and outer surfaces of the fiber were mounted on the specimen stub using carbon tape and then sputter-coated with a gold layer for 300 s using an Edwards Scancoat Six Sputter Coater.

**Fig. 7:**
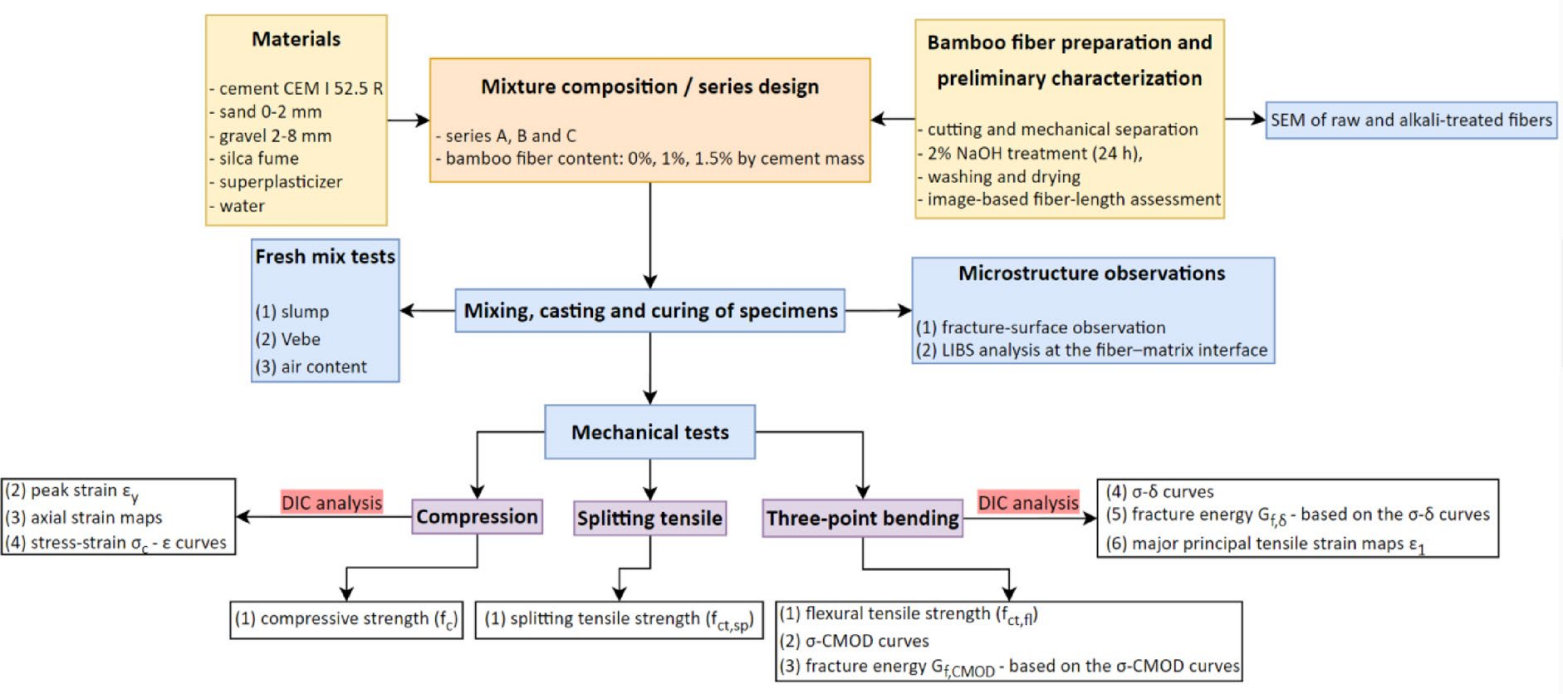
Schematic overview of the experimental program and experimental methodology.

Figure 7 presents a schematic overview of the experimental program and the main stages of the experimental procedure described in this section.

## Results and discussion

### Fresh mix properties

Table 2 summarizes the test results for mixtures from Series A–C, including air content (A_air_), slump (F_T_), and Vebe time (V_B_). For each parameter, the mean value $\left( {{\bar{\mathrm{x}}}} \right)$, standard deviation (s), and coefficient of variation (v) were calculated. The values ${{\bar{\mathrm{x}}}} \pm \Delta$ denote the half-width of the two-sided 95% Student’s t-confidence interval (as explained in the table footnote). Owing to the small sample size, comparisons between series were performed using a non-parametric bootstrap procedure (10.000 replications; resampling with replacement within each series), and 95% percentile confidence intervals (CI) were determined for the differences between means. Differences were considered significant when the 95% CI did not include zero.

The addition of bamboo fibers (Series B and C) changed the properties of the fresh mix. In terms of consistency, a reduction in F_T_ slump of approximately 15–17 mm and an increase in Vebe time of approximately 2–4 s were recorded. In contrast, the air content A_air_ increased by approximately 1.2–1.5% points. The bootstrap analysis showed statistically significant differences in mean values for A_air_ and V_B_ in all pairwise comparisons between series, whereas for F_T_ no significant difference was found between Series B and C (the 95% CI for Series C–B included 0; −3 to 0 mm). All mixtures were classified as consistency class S1 (slump) and V2 (Vebe).

The obtained results for A_air_, F_T_, and V_B_ are consistent with literature reports indicating that fiber addition is often associated with an increase in air content and a deterioration in mix consistency^1,28,30^. This may be attributed to the physical effect of fibers on mix flow, as the fibers act as barriers that hinder the movement of the cement paste and increase internal resistance^32^. At higher fiber contents, the total fiber–paste contact surface also increases, which raises the water demand required for fiber wetting^30^. Under such conditions, compaction and deaeration of the mix become more difficult, which may promote the retention of air voids in the mixture^1^.

**Table 2:** Fresh-mix test results (definitions of the symbols are provided in the text).

s. No	A_air_	F_T_	V_B_
	%	mm	s
Series A	2.4	35	14
	2.4	24	13
	1.8	29	14
${{\bar{\mathrm{x}}}}$	**2.2 ± 0.9**	**29.3 ± 13.7**	**13.7 ± 1.4**
**s**	**0.3**	**5.5**	**0.6**
**ν**	**15.7%**	**18.8%**	**4.2%**
Series B	3.4	15	17
	3.4	14	16
	3.3	13	15
${{\bar{\mathrm{x}}}}$	**3.4 ± 0.1**	**14 ± 2.5**	**16 ± 2.5**
**s**	**0.1**	**1.0**	**1.0**
**ν**	**1.7%**	**7.1%**	**6.3%**
Series C	3.6	14	18
	3.8	11	18
	3.6	12	17
${{\bar{\mathrm{x}}}}$	**3.7 ± 0.3**	**12.3 ± 3.8**	**17.7 ± 1.4**
**s**	**0.1**	**1.5**	**0.6**
**ν**	**3.1%**	**12.4%**	**3.3%**

$${{\bar{\mathrm{x}}}} \pm \Delta$$ $${\mathrm{where}}\:\Delta \: = {\mathrm{t}}_{{0.975,{\mathrm{n}} - 1}} \frac{s}{{\sqrt {\mathrm{n}} ,}},$$

n-number of specimens

Bold values indicate the descriptive statistical measures for each series, namely the mean (${{\bar{\mathrm{x}}}}$), standard deviation(**ν**), and coefficient of variation (**ν**).

### Mechanical properties

Table 3 summarizes the results of the material characteristics: compressive strength (f_c_), ultimate strain (ε_y_) corresponding to the attainment of the maximum stress in the compression test, splitting tensile strength (f_ct, sp_), and flexural tensile strength (f_ct, fl._). Fracture energy values (G_f,δ_ and G_f, CMOD_) are also provided. The statistical symbols (${{\bar{\mathrm{x}}}}$¯, s, and ν) correspond to the values described in Table 2.

The flexural tensile strength was determined according to Eq. (1), assuming the maximum load (F_max_). Fracture energy G_f_ was determined in two variants, according to Eqs. (2) and (3), using two displacement measures: deflection δ and CMOD. Deflection δ represents a global measure of specimen response, whereas CMOD refers directly to crack opening in the notch zone. The use of both variants results from the fact that δ and CMOD are regarded as complementary measures for describing the fracture kinematics of notched beams^54,55^. The value of the integral was estimated numerically using the rectangle method.

1 $$\:\sigma\:=\frac{3Fl}{2b{\left(h-{a}_{0}\right)}^{2}}$$

2 $$\:{G}_{f,\delta\:}=\frac{h}{h-{a}_{0}}{\int\:}_{0}^{{\delta\:}_{u}}\sigma\:d\delta$$

3 $$\:{G}_{f,CMOD}=\frac{h}{h-{a}_{0}}{\int\:}_{0}^{{CMOD}_{u}}\sigma\:dCMOD$$

where:

F, l, b, h, a_0_ - for the beam: load, span, width, height, and notch depth (initial notch), respectively; δ_u_ and CMOD_u_ - the terminal values of deflection and CMOD adopted for integration (recorded during the test).

**Table 3:** Material characteristics (definitions of the symbols are provided in the text).

Range bamboo fibers	f_c_	ε_y_	f_ct, sp_	f_ct, fl._	G_f,δ_	G_f, CMOD_
0% series A	MPa	‰	MPa	MPa	J/m^2^	J/m^2^
	88.2	2.31	4.92	5.77	152.3	114.8
	85.9	2.32	5.40	5.17	128.6	134.0
	87.8	2.38	5.42	4.88	109.1	94.5
	75.4	1.91	3.68			
	77.4	2.13	4.52			
${{\bar{\mathrm{x}}}}$	**82.9 ± 7.6**	**2.21 ± 0.24**	**4.79 ± 0.90**	**5.28 ± 1.13**	**130 ± 53.7**	**114.4 ± 49.0**
**s**	**6.1**	**0.19**	**0.72**	**0.45**	**21.6**	**19.7**
**ν**	**7.4%**	**8.8%**	**15.1%**	**8.6%**	**16.6%**	**17.2%**
1% series B	102.6	2.48	5.09	6.15	166.8	150.1
	84.5	2.31	5.12	5.91	149.9	123.1
	83.3	2.20	3.92	5.64	151.9	138.2
	89.0	2.74	4.43			
	88.9	2.60	4.95			
${{\bar{\mathrm{x}}}}$	**86.4 ± 4.7**	**2.46 ± 0.40**	**4.7 ± 0.64**	**5.9 ± 0.63**	**156.2 ± 22.9**	**137.1 ± 33.5**
**s**	**2.9**	**0.25**	**0.52**	**0.25**	**9.2**	**13.5**
**ν**	**3.3%**	**10.2%**	**11.0%**	**4.3%**	**5.9%**	**9.8%**
1.5% series C	80.2	2.68	3.99	6.02	167.1	120.4
	77.4	2.27	4.28	5.92	425.5	151.0
	77.0	1.95	4.05	5.62	354.5	186.5
	79.1	2.28	3.93			
	74.6	2.68	4.33			
${{\bar{\mathrm{x}}}}$	**77.6 ± 2.7**	**2.37 ± 0.38**	**4.12 ± 0.22**	**5.86 ± 0.51**	**315.7 ± 331.5**	**152.6 ± 82.2**
**s**	**2.1**	**0.31**	**0.18**	**0.21**	**133.5**	**33.1**
**ν**	**2.8%**	**13.1%**	**4.4%**	**3.5%**	**42.3%**	**21.7%**

${{\bar{\mathrm{x}}}} \pm \Delta$denotes the mean and the half-width of the two-sided 95% Student’s t confidence interval, where $\:{\Delta\:}={\mathrm{t}}_{0.975,\mathrm{n}-1}\mathrm{s}/\sqrt{\mathrm{n}}$.

Bold values indicate the descriptive statistical measures for each series, namely the mean (${{\bar{\mathrm{x}}}}$), standard deviation(**ν**), and coefficient of variation (**ν**).

**Fig. 8:**
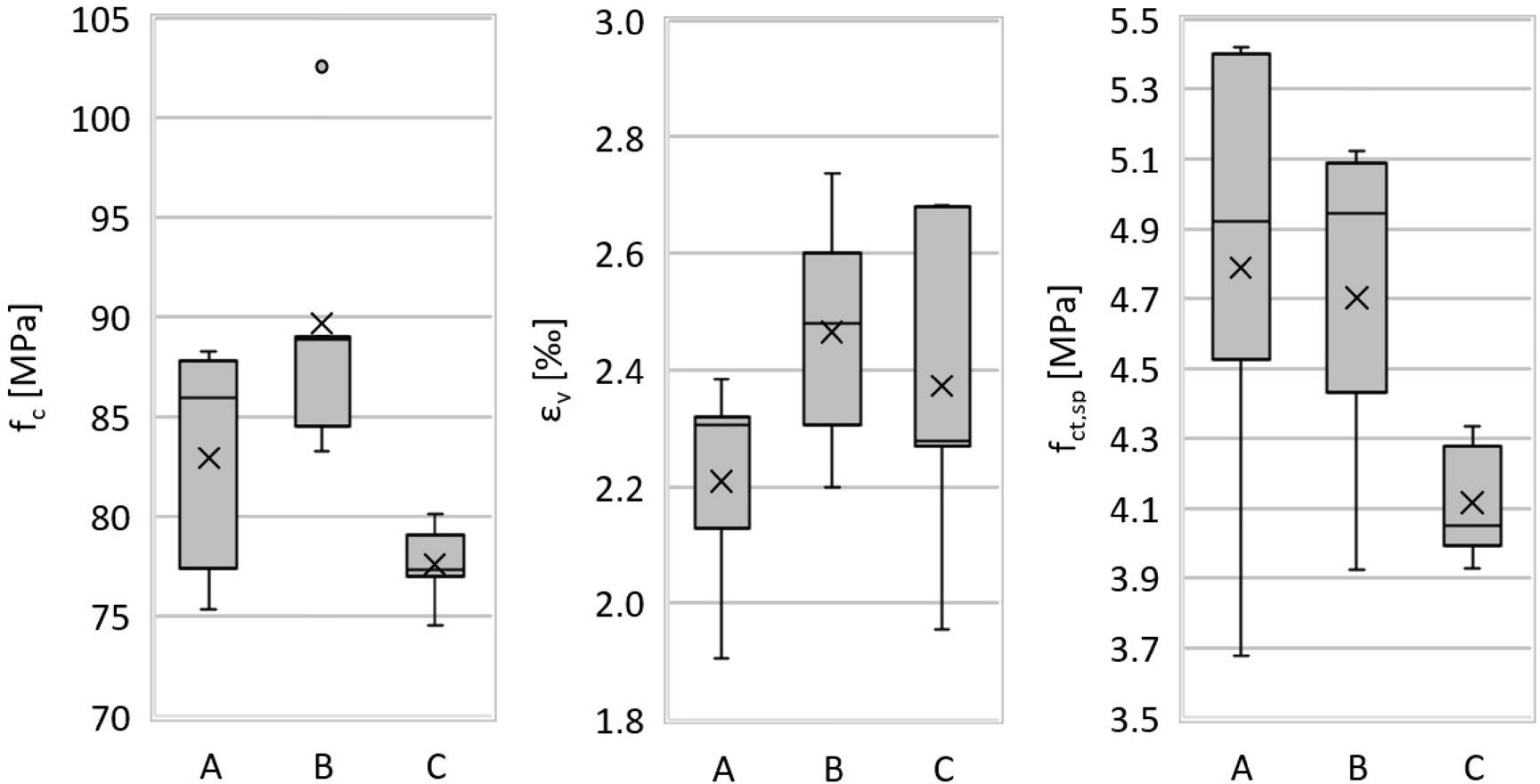
Box-and-whisker plots for compressive strength (f_c_), strain corresponding to the maximum compressive stress (ε_y_), and splitting tensile strength (f_ct, sp_) for Series A (0%), B (1%), and C (1.5%).

Selected measures of central tendency and dispersion for f_c_, ε_y_, and f_ct, sp_ are presented as box-and-whisker plots in Fig. 8. The plots show mean values (marked with the “×” symbol), medians, quartiles (Q1 and Q3), the non-outlying range, and outliers identified using the 1.5 × IQR criterion, where IQR (interquartile range) denotes the difference between Q3 and Q1. The highest mean value of fc was recorded for Series B, with a single outlier observation also identified (102.6 MPa), which was excluded from further analyses. Series A exhibited the largest interquartile range, whereas Series C showed the lowest mean value and the smallest variability. For ε_y_, the highest central values (median and mean) occurred in Series B, whereas Series A and C had lower values, with Series C showing greater dispersion. For f_ct, sp_, the central values in Series A and B were comparable, whereas Series C was characterized by a lower value and a narrower interquartile range.

The results indicate that the effect of bamboo fiber addition on f_c_ depended on the dosage level. In Series B (1%), the mean f_c_ value was 4.2% higher than that of the reference series, whereas in Series C (1.5%) a decrease of 6.4% was recorded. One-way ANOVA showed a significant effect of series (*p* < 0.05), while Tukey’s post hoc test confirmed a significant difference only between Series B and C. The reduction in compressive strength observed in Series C for high-strength concrete modified with bamboo fibers may result from unfavorable differences between the strength properties and elastic modulus of the fibers and the properties of the cementitious matrix^34^, the presence of air voids in the composite structure^56^(see Table 2), and weakened adhesion at the matrix–aggregate interface^4,30^. The literature reports decreases in compressive strength of up to 24% for fiber volume contents ranging from 0 to 3%, compared with unreinforced concrete^6,20,30,34,57^. In contrast, other studies found that the addition of fibers at 1% and 1.5% relative to cement mass increased compressive strength by 8–14%^41^, whereas Ref.^40^ reported a 12% decrease in the compressive strength of high-strength concrete. Other studies have reported increases ranging from 2% to 32%^2,58,59^, which have been attributed to the crack-bridging mechanism, leading to energy absorption and delayed crack propagation^1,4^.

For the strain at maximum compressive stress, ε_y_, increases in mean values relative to the reference series were recorded: 11.3% in Series B and 7.2% in Series C, after excluding the outlier value of 2.48‰. These differences were not statistically significant (ANOVA, *p* > 0.05), which may partly be attributed to the considerable variability of the results within the groups. The ε_y_ values were characterized by relatively wide interquartile ranges (Series B and C), which reduced the statistical power of the test. This variability may result from local differences in matrix microstructure, including the distribution of voids and fibers^6^.

Likewise, no statistically significant differences between series were found for the splitting tensile strength f_ct, sp_ (ANOVA, *p* > 0.05). Relative to the reference series, the mean f_ct, sp_ value in Series B was 1.9% lower, whereas in Series C it was 14% lower. The literature reports increases in f_ct, sp_ of 1.2%^58^, 40%^6^, and 79%^28^ for fiber volume contents up to 1%. In turn, the addition of fibers at 1% and 1.5% relative to cement mass increased the f_ct, sp_ of high-strength concrete by 8–44%^41^ and by 36%^40^. Other studies^57^ reported a decrease in f_ct, sp_ of approximately 15% for bamboo fiber additions of 1–1.5% relative to cement mass, as well as a 32% decrease^34^ for bamboo fiber volume contents of 4–8%. These discrepancies may result from differences in the type and geometry of the fibers used, the properties of the fresh mix, and the casting conditions, which affect the distribution of fibers within the matrix, including their tendency to agglomerate^1^. These factors may jointly influence the tensile response of the composite by affecting the conditions of crack initiation and development^60^.

Unlike f_ct, sp_, the addition of bamboo fibers increased the flexural tensile strength f_ct, fl._ relative to the reference series by approximately 12% in Series B and approximately 11% in Series C. The significance of the mean differences was assessed analogously to Sect.  3.1, using 95% confidence intervals for the mean differences. The mean strength of Series B was higher than that of Series A by 0.63 MPa (0.13–1.09 MPa), and higher than that of Series C by 0.58 MPa (0.09–1.01 MPa). In both cases, the intervals did not include zero. The difference between Series B and C was 0.05 MPa, and its 95% CI (-0.26 to 0.35 MPa) included zero, indicating no significant difference between these groups. Literature data are inconclusive: at a bamboo fiber volume content of 1%, increases in concrete f_ct, fl._ of 5–57%^6,28,58^, a decrease of 25%^59^, or no effect^30^ have been reported. These discrepancies are attributed, among other factors, to differences in fiber geometry and orientation, as well as to changes in mix porosity^1^.

An analogous analysis was carried out for the fracture energy of the composite determined from the bending curves, namely G_f,δ_ and G_f, CMOD_. Relative to the reference series, G_f,δ_ increased by 20.2% in Series B and by 142.8% in Series C, with mean differences of 26.2 J/m2 (4.6–47.1) and 185.7 J/m2 (38.5–292.7), respectively. For G_f, CMOD_, the increase was 19.8% in Series B and 33.4% in Series C, and the corresponding mean differences were 22.7 J/m^2^ (0.5–44.9) and 38.2 J/m^2^ (3.0–73.4). Since the confidence intervals for the differences did not include zero, the results indicate a statistically significant increase in fracture energy G_f,δ_ and G_f, CMOD_. The higher fracture energy values in Series C relative to Series A may be associated with delayed initiation of microcracking in the notch-tip zone and short-term load retention near the maximum value, which translated into slightly higher recorded values of δ and CMOD. This issue is discussed in greater detail in Sect.  3.3. The observed increase in composite fracture energy with increasing fiber content is partly consistent with literature data^4,31^, although those results concerned lightweight aggregate concretes and mortars.

The mean density of specimens in the tested series was as follows: A – 2298 kg/m^3^, B – 2271 kg/m^3^, and C – 2268 kg/m^3^. The addition of bamboo fibers was associated with a reduction in composite density of approximately 1.2–1.3% relative to the reference series, which is consistent with literature reports^34^. This effect may be associated with the lower density of the fibers compared with the cementitious matrix and aggregate, as well as with a potential increase in the proportion of pores and entrapped air around the fibers, including within the interfacial zone, resulting from the rough and hygroscopic surface of the fibers^1^.

### Stress–strain (compression) and stress–deflection / stress–CMOD (bending) curves with DIC full-field strain analysis

**Fig. 9:**
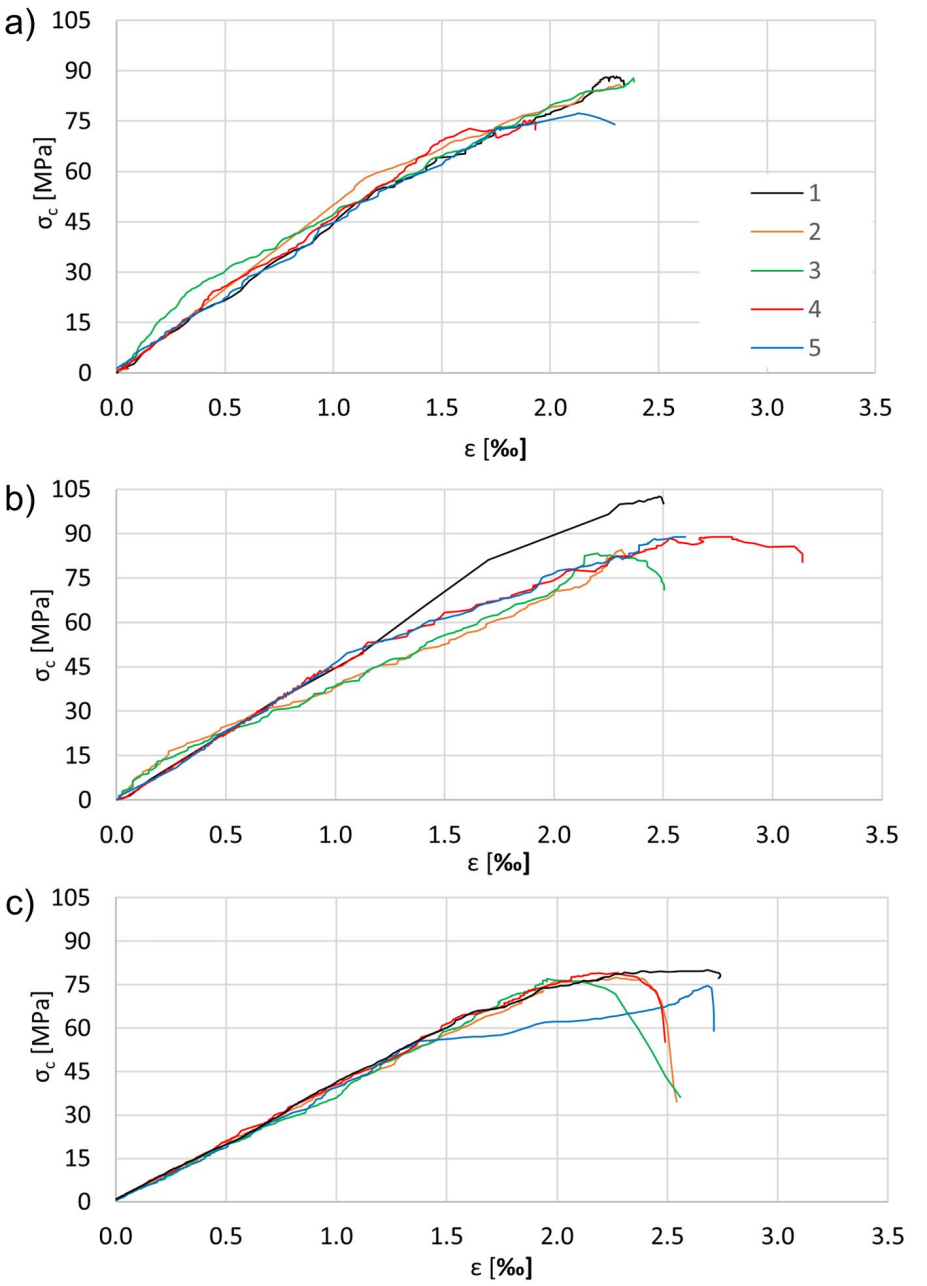
σ_c_–ε curves obtained in compression for the individual series: (**a**) A; (**b**) B; (**c**) C (legend: numbers denote specimen number).

**Fig. 10:**
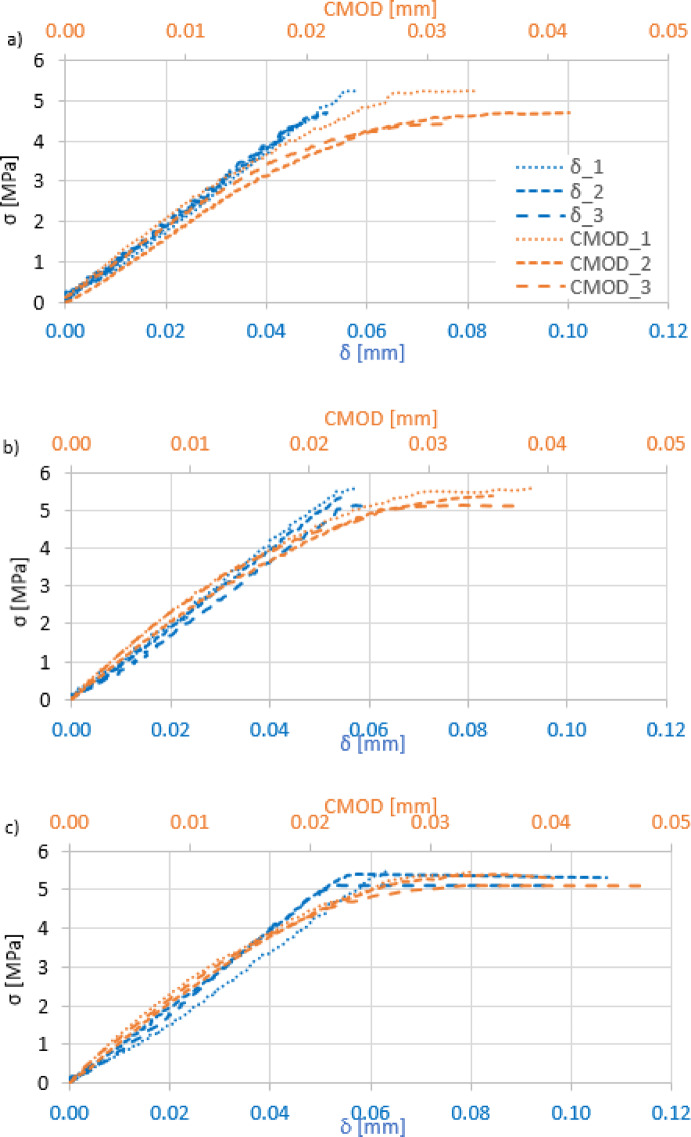
σ–δ and σ–CMOD curves of beams tested in three-point bending: (**a**) series A; (**b**) series B; (**c**) series C (legend: blue lines - σ–δ; orange lines - σ–CMOD; numbers denote beam number).

**Fig. 11:**
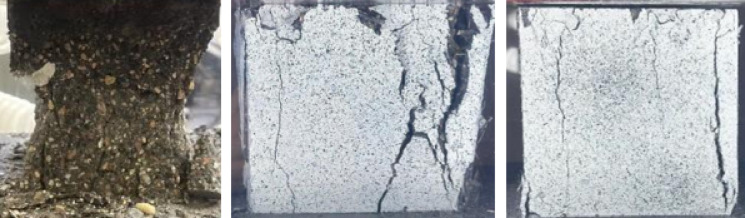
Example images of specimen damage after the compressive strength test (from left: series A, B, and C): (**A**)-conical failure; (**B**)-dominant diagonal crack; (**C**)-dominant approximately vertical cracking/fracture.

**Fig. 12:**
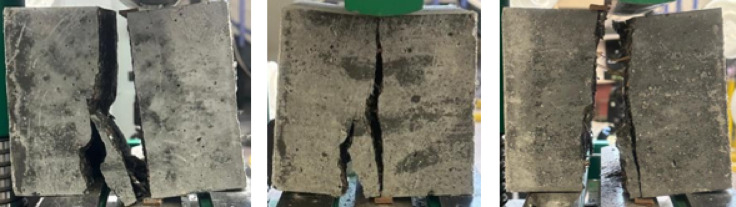
Example images of damage in concrete specimens after the splitting tensile strength test (from left: series A, B, and C): (**A**)-single crack; (**B**)-partial separation; (**C**)-complete separation with visible fibers.

**Fig. 13:**
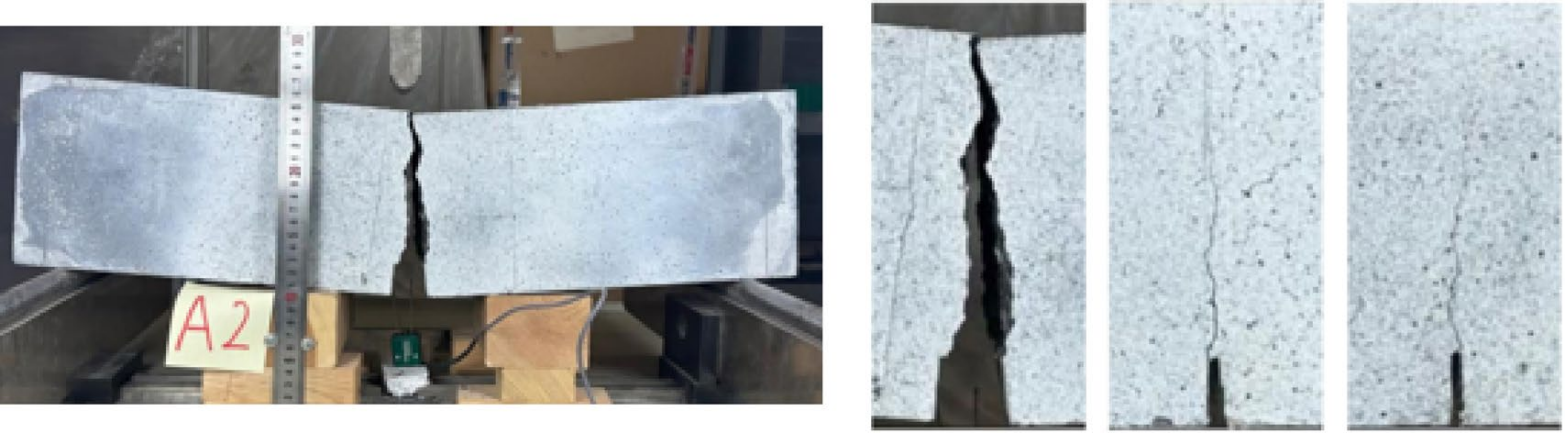
Example images of beam failure after three-point bending: (from left) beam A2 and close-ups of the notch region for selected beams from series A, B, and C, showing differences in crack extent and crack opening.

Figure 9 shows the σ_c_–ε curves of specimens under compression for different bamboo fiber contents (Series A, B, and C). Similarly, Fig. 10 presents the stress–deflection (σ–δ) curves and the stress–crack mouth opening displacement (σ–CMOD) curves for beams tested in three-point bending. Representative images of damaged specimens after compression, splitting tensile, and bending tests are shown in Figs. 11, 12 and 13.

Analysis of the σ_c_–ε curves for specimens in compression in all series indicates a similar slope of the approximately elastic portion, which suggests that the introduction of fibers did not cause substantial changes in the initial stiffness of the composite. In Series A, after reaching the maximum compressive stress σ_c, max_ (Fig. 9a), a rapid stress drop and limited development of inelastic strains are observed, which is typical of concretes with elevated strength, including high-strength concretes^52^. In Series B and C, the post-peak portions of the specimen curves are generally extended, show a more gradual decrease, and cover a wider range of recorded strains after σ_c, max_; however, the final stress drop remains essentially abrupt (Fig. 9b–c). These results indicate that, for the applied fiber dosage and preparation procedure, the effect of the fibers on the response after exceeding σ_c, max_^1^ was limited, and the failure mode remained predominantly quasi-brittle. Similar trends in the σ_c_–ε curves under compression have also been reported for high-strength concretes modified with other natural fibers, such as coconut fibers^61^.

In the compression and splitting tests, specimens from Series A failed abruptly, with clear separation of fragments (Figs. 11 and 12). In Series B and C, the presence of fibers promoted the retention of fragment integrity after failure and limited their complete separation.

For beams from all series, the σ–δ and σ–CMOD relationships were initially approximately linear-elastic (Fig. 10). The differences between the σ–δ and σ–CMOD curves may be explained by the fact that CMOD is a local measure of crack opening at the notch, sensitive to crack initiation and propagation, whereas deflection δ also includes the global compliance of the test setup, such as rotations and shear effects. Consequently, nonlinearity may become apparent over different ranges of the two measures^55^.

In Series A, the σ–δ curves remained quasi-linear up to the maximum load, whereas the σ–CMOD curves showed flattening in the region of the highest stresses (Fig. 10a). In Series B, the shape of the curves remained generally similar to that of the reference series, indicating a limited effect of the 1% fiber addition on beam response in the vicinity of the maximum load (Fig. 10b). In Series C, a wider range of δ and CMOD was observed near the maximum load; however, after this point, a sudden loss of the capacity to sustain further load occurred (Fig. 10c).

The effect observed in Series C may be associated with delayed initiation of microcracking in the notch-tip zone resulting from the presence of fibers^60^. The limitation of microcrack initiation and coalescence promoted short-term load retention near the maximum value, which translated into slightly higher recorded values of δ and CMOD. However, the obtained results do not confirm a distinct ability of the composite to sustain load after exceeding the peak load, as described in some studies as an effect of the strengthening action of fibers in this phase^1,34^. Literature reports indicate that a more gradual descending branch of the σ–δ curve and a more pronounced bridging effect are usually observed at higher bamboo fiber dosages (4–16% by mass or 4–8% by volume)^34,35,62^, whereas at lower dosages (0–2% by volume) this effect is usually limited and leads at most to low residual stresses in the composite^26^.

**Fig. 14:**
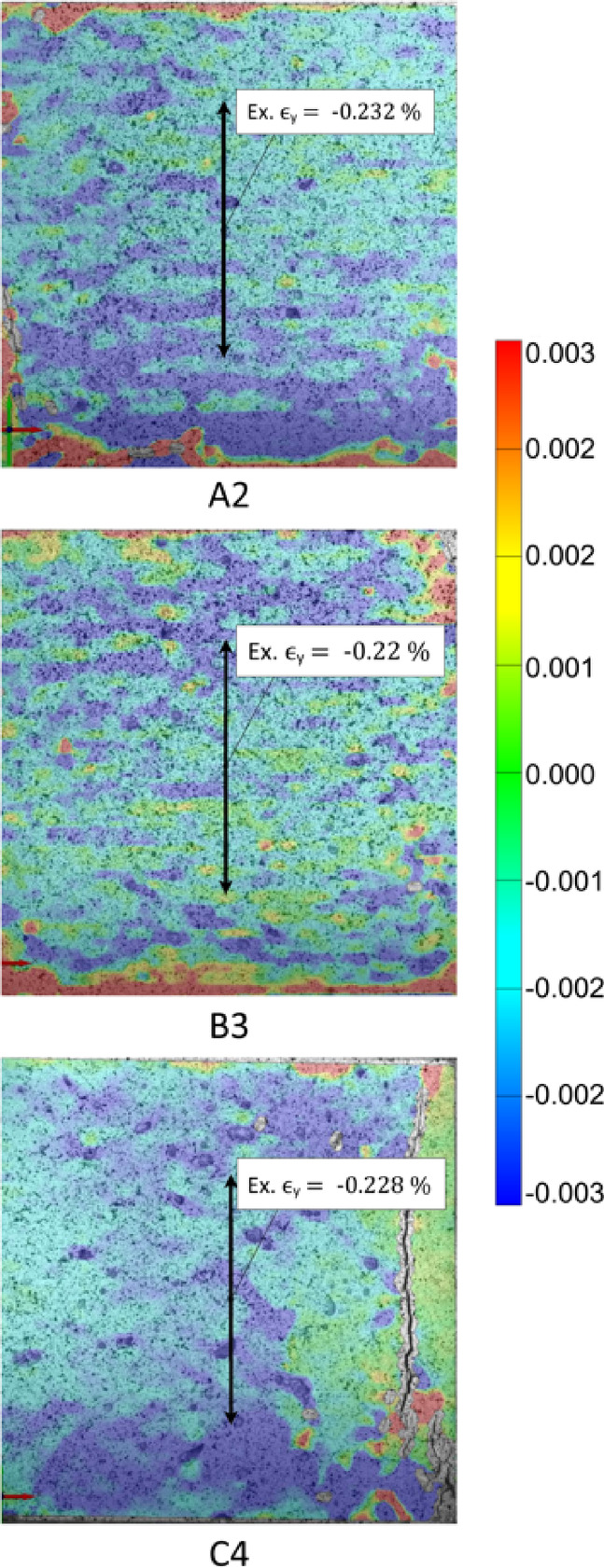
Axial strain ε_y_ (DIC) maps for selected specimens from series (**A**)–(**C**) at σ_c, max_. The color scale shows ε_y_ (dimensionless), and the black line denotes the virtual extensometer (ε_y_ in %).

Figure 14 presents representative maps of compressive strain ε_y_ for selected specimens A2, B3, and C4, one from each series, recorded by DIC at σ_c, max_, together with the ε_y_ values determined from the virtual extensometer (Ex.; Fig. 6). Despite similar strain values recorded over the extensometer gauge length, the εy maps do not indicate a uniform field distribution over the entire observed surface. In the central parts of the specimens, the strain distribution is more even, whereas local field disturbances are visible near the edges. In specimens A2 and B3, near-edge zones with lower values of compressive strain ε_y_ are visible and are marked by red-orange colors. Their location is consistent with the effect of specimen contact conditions with the machine platens, which is typical of compression tests, where the restraint of lateral strains leads to local disturbance of the strain field in zones adjacent to the loaded surfaces^63^. A different pattern is observed in specimen C4, in which a crack is visible near the right edge. In its vicinity, a local reduction in compressive ε_y_ is observed relative to the remaining part of the specimen, indicating clear asymmetry of the strain field. Thus, comparison of the virtual extensometer readings with the DIC maps shows that similar ε_y_ values over the gauge length do not imply an identical strain distribution over the entire observed specimen surface. At the same time, the presented maps of selected specimens do not allow an unambiguous effect of bamboo fibers on the ε_y_ distribution at σ_c, max_ to be attributed.

**Fig. 15:**
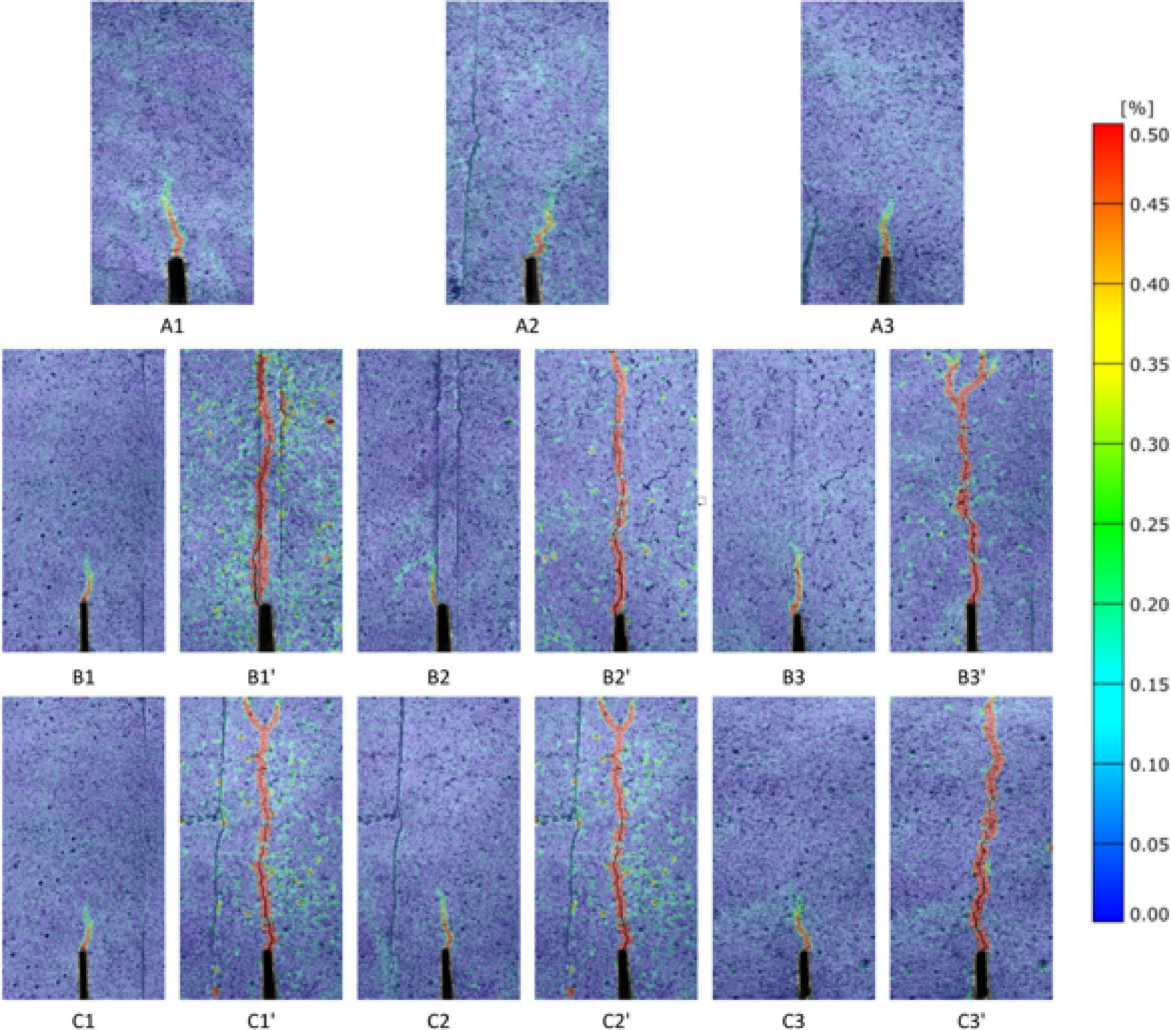
Maps of principal tensile strain ε_1_ (DIC) in the notch region of bent beams. Series (**A**)–(**C**), beams 1–3 (described in the text).

In DIC analysis, principal tensile strain fields are used to identify strain-localization zones associated with crack development in notched elements^31,64^. For this reason, Fig. 15 presents ε_1_ maps in the notch region of the beams. Images without a prime correspond to the state at the maximum beam load, whereas images marked with a prime correspond to the state immediately after this load was reached. Such a comparison makes it possible to assess the change in the extent of the ε_1_ localization zone during the transition from maximum load to further crack development.

At maximum load, the length of the zone with elevated ε_1_ values, measured from the notch tip, averaged 31.05 mm for Series A, 22.54 mm for Series B, and 23.39 mm for Series C, corresponding to reductions of 27.4% and 24.7%, respectively, relative to the series without fibers. This means that in the fiber-reinforced series, the strain localization state at maximum load remained more concentrated in the immediate vicinity of the notch than in the series without fibers. Immediately after the maximum load was reached, the ε_1_ zone extended upward along the beam, following a curvilinear trajectory consistent with further crack development. When considered together with the σ–δ and σ–CMOD curves and the fracture energy results, this observation indicates that, in the fiber-reinforced series, crack development in the notch region was delayed relative to the series without fibers, whereas the contribution of the fibers after the maximum load had been reached was short-lived. The local gray regions visible on some maps were treated as effects of measurement and visualization limitations, that is, artifacts of the DIC method, and were not used as a basis for interpreting the course of ε_1_ localization.

### Microstructure

**Fig. 16:**
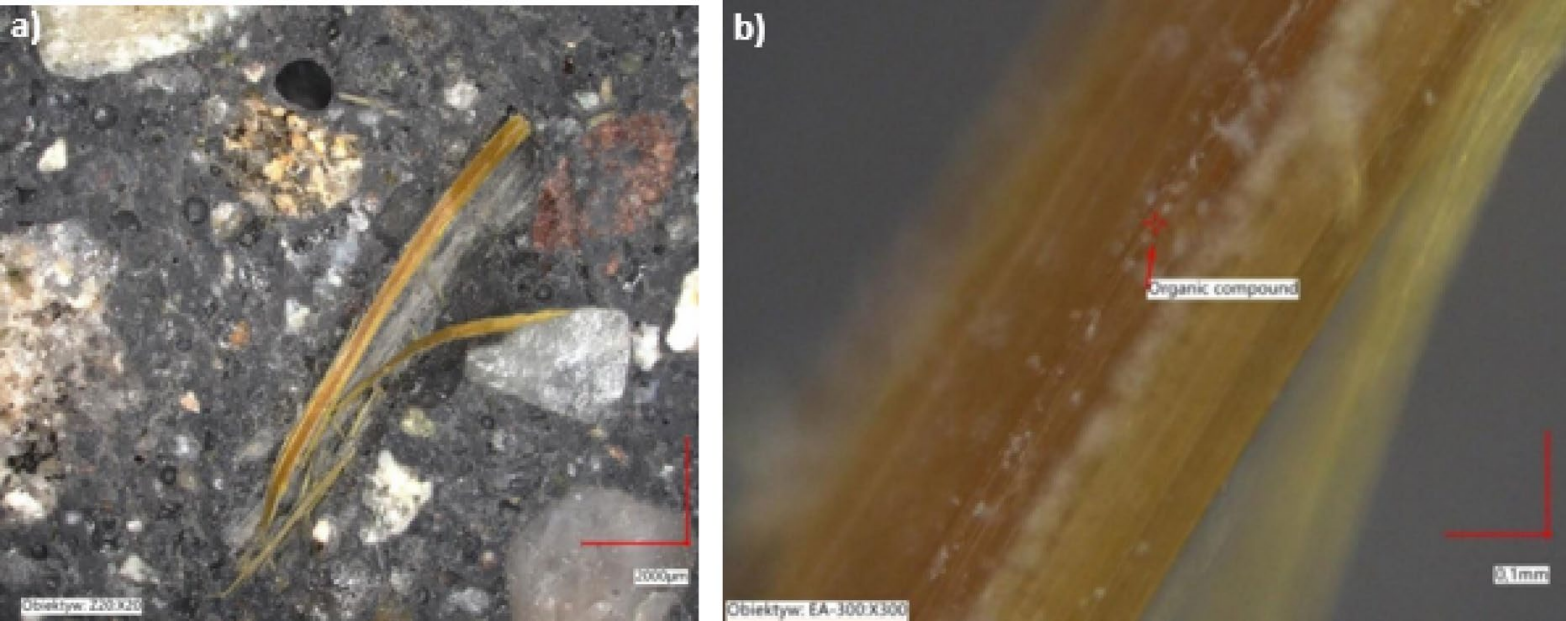
Images of a bamboo fiber visible on the fracture surface of a specimen after the splitting tensile strength test: (**a**) general view of the area with the fiber embedded in the matrix; (**b**) close-up of the fiber surface in the fiber–matrix contact region.

Figure 16 shows images of a bamboo fiber located in the cement matrix on the fracture surface of a specimen after the splitting tensile strength test. The visible local delamination at the fiber ends indicates that the fiber was damaged during composite failure (Fig. 16a). However, the magnified view of the fiber surface (Fig. 16b) does not reveal, at the optical scale, any unambiguous morphological features that could be reliably associated with a significant surface modification resulting from the applied alkali treatment (2% NaOH, 24 h). Therefore, this figure should be regarded primarily as qualitative documentation of the presence of the fiber in the fracture zone and of its condition after specimen failure.

**Fig. 17:**
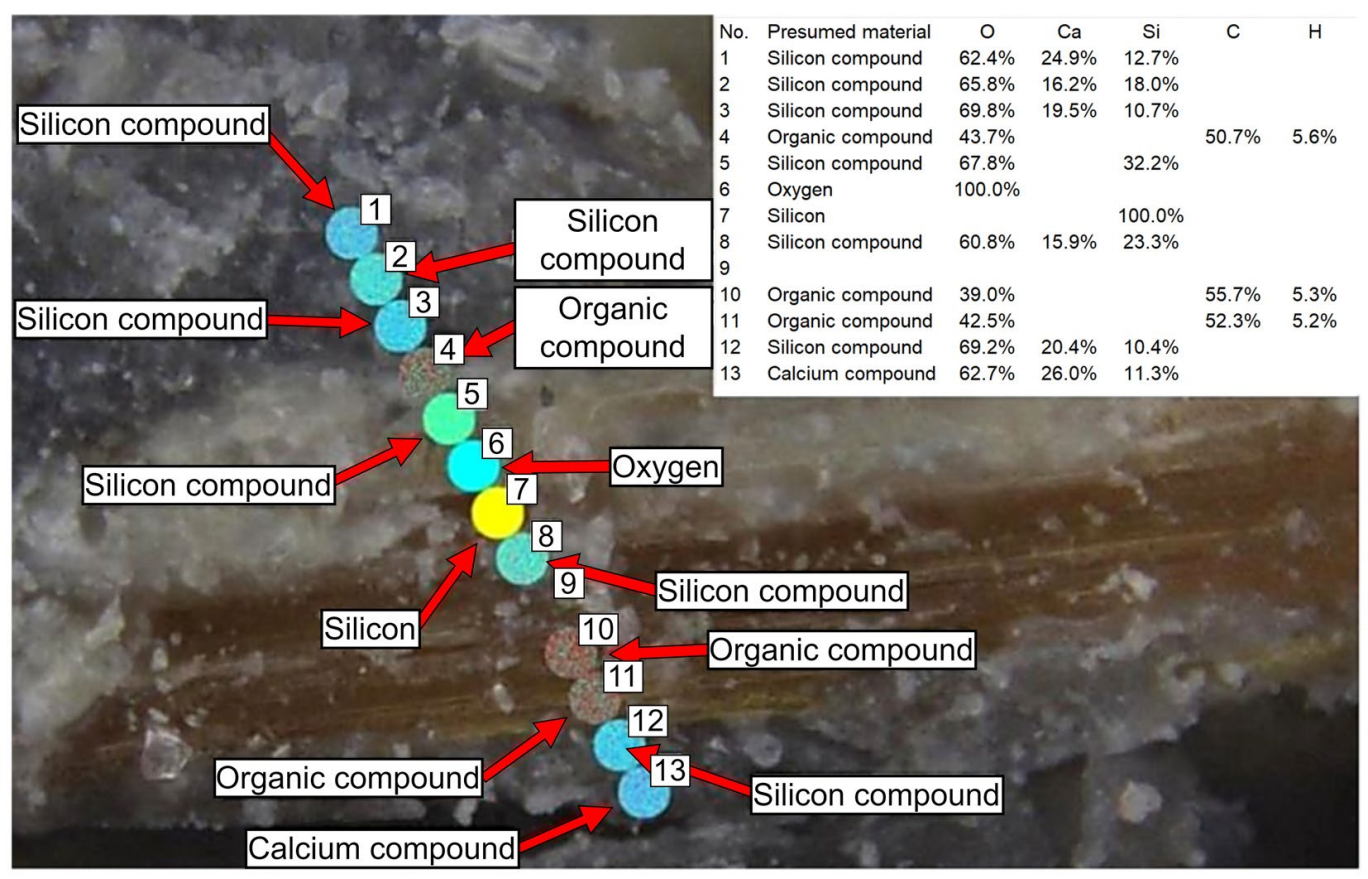
Pointwise LIBS elemental analysis along the fiber–matrix boundary on the fracture surface. The measurement points and their classification (organic/mineral) are indicated, together with a summary of the recorded elemental signals.

To localize the fiber–matrix boundary and qualitatively assess the material contrast in its immediate vicinity, point elemental analysis along the interface was performed using the LIBS method (Fig. 17). Points located within the fiber were classified as organic regions, whereas points located in the matrix were classified as mineral regions, which confirmed a change in material character at the phase boundary within the analyzed microregion. Due to the point-based nature of the measurement and the irregularity of the fracture surface, the LIBS results were interpreted qualitatively. In this sense, Figs. 16 and 17 provide complementary qualitative characterization of the fiber–matrix contact zone and form a basis for further observations carried out using SEM.

**Fig. 18:**
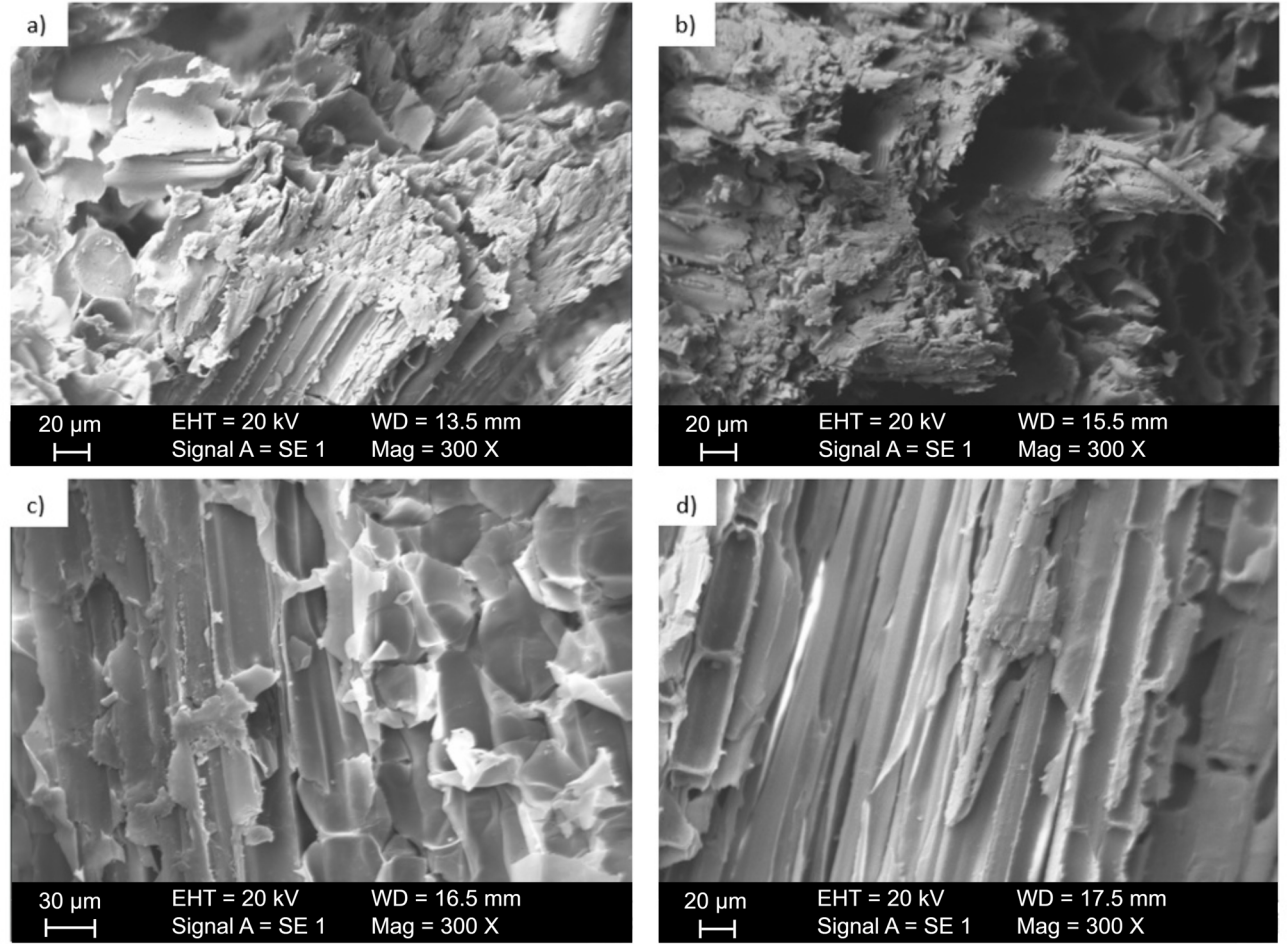
SEM images of bamboo fibers in the raw state (RAW, left column) and after alkali treatment (2% NaOH, 24 h, right column): (**a**–**b**) cross-sectional views; (**c**–**d**) longitudinal surface views of the fiber.

SEM images of bamboo fibers in the raw state (RAW) and after alkali treatment are presented in Fig. 18. In the cross-sectional views, both cases show local delamination of the fibrous structure and an irregular fracture pattern (Fig. 18a–b). In the views of the longitudinal fiber surface (Fig. 18c–d), both the RAW fibers and the NaOH-treated fibers exhibit a banded and grooved morphology consistent with the fiber structure, with local fragmentation and delamination. In the image after NaOH treatment, more continuous and elongated bands are visible in some areas (Fig. 18d), whereas in the RAW state, irregular flake-like fragments are locally present (Fig. 18c). However, these differences are qualitative in nature and do not allow an unambiguous confirmation of a distinct surface morphology modification after alkali treatment. On this basis, the changes in fiber surface features at the scale assessed in the SEM images should be regarded as at most limited, which may explain the slight change in the mechanical properties of the composite with fibers relative to the reference series (f_c_, f_ct, sp_, f_ct, fl._).

At the same time, the literature indicates that more intensive alkali treatment, at concentrations above 2%, may more effectively remove amorphous constituents and surface impurities, including hemicellulose and lignin, potentially increasing surface development and the possibility of interactions at the fiber–matrix interface^20,21,23,65,66^. However, the effectiveness of such modification depends on many factors, including fiber origin and preparation procedure, which is reflected in the discrepancies reported in the literature. Some studies used alkali treatment with a solution concentration of approximately 2% NaOH^1,2,5^, whereas others did not apply alkali treatment at all^4,11,27^. Another possible way to increase interfacial adhesion between fiber and matrix is the application of silane treatment^67,68^ or sodium hydroxide treatment assisted by microwaves and hot water^19,21^. For this reason, further studies may include a comparison of several fiber modification variants and the use of complementary techniques, such as µXRT, FTIR, and TEM^26^, allowing a more comprehensive characterization of chemical and morphological changes and their relationship with the mechanical response of the composite.

### Comparative discussion in the context of previous studies

With regard to the bending of notched beams, the obtained results^69^ indicate that, for the applied bamboo fiber geometry, dosage, and alkali treatment, no distinct crack-bridging effect was identified. At the same time, a significant increase was found for both measures of fracture energy, G_f,δ_ and G_f, CMOD_. When considered together with the σ–δ and σ–CMOD curves and the ε_1_ maps, this indicates delayed initiation of microcracking in the notch-tip zone, limited microcrack coalescence, and short-term load retention near the maximum value. These observations differ from those reported in some studies, in which the contribution of bamboo fibers after matrix cracking was more pronounced^4,26,31^, but remain consistent with the observation that the increase in energy dissipation associated with crack propagation may result not only from load transfer by fibers crossing the crack and stabilizing it (fiber bridging), but also from weakening and failure of the fiber–matrix bond (fiber debonding), fiber pull-out, and fiber rupture^70^. The variability of results reported in the literature may be related to the fact that the influence of fibers on the mechanical properties and fracture parameters of the composite depends on many interacting factors, including the quality of the fiber–matrix interface^1,2^, fiber geometry^34^, the mechanical properties of bamboo^15^, fiber content^1^, and the preparation procedure^71^.

Studies on the hardened properties of cementitious composites modified with materials of reduced environmental impact have been the subject of numerous investigations due to the need for sustainable development. The literature addresses, among other factors, the effects of fly ash^34^, limestone powder^2^, silica fume^42,69,72,73^, tire-cord fibers^74^, and aggregate type^38,75^ on selected mechanical properties of the composite, and in some studies also on fracture parameters. Against this background, data on high-strength concrete reinforced with bamboo fibers remain limited, particularly with regard to the quantitative assessment of compressive deformability. In the present study, the addition of fibers did not produce a distinct change in the post-peak portion of the σ_c_–ε curve in compression, whereas after failure a greater integrity of specimen fragments was observed compared with specimens without fibers. In addition, the analysis of compressive strain maps ε_y_ made it possible to identify asymmetry and local gradients in the strain field resulting from the boundary conditions of the test and from structural discontinuities within the specimens. The possibility of identifying such phenomena is substantially limited when traditional point-based measurement methods are used^76,77^.

With reference to the literature, the present study confirmed that the effect of bamboo fiber addition on the analyzed fresh-mix properties and on the parameters characterizing the mechanical response of the composite was diverse^1^. An important complement to this assessment was the analysis of the statistical significance of the observed changes, which made it possible to distinguish effects associated with the presence of fibers from differences falling within the range of result scatter.

## Conclusions

Based on the presented test results and the analyses performed, the following conclusions were formulated:

- The addition of bamboo fibers caused statistically significant changes in the fresh-mix properties, including an increase in air content by approximately 55–68% and a deterioration in consistency, expressed by a decrease in slump of approximately 52–58% and an increase in Vebe time. These results may be associated with hindered mix flow and more difficult compaction and deaeration due to the presence of fibers.
- The effect of bamboo fiber addition on the mechanical properties of concrete was diverse. Relative to the reference series, the f_c_ value increased by 4% at a fiber content of 1.0% and decreased by 6% at 1.5%, while statistical significance was confirmed only between these dosage levels. For f_ct, sp_, no statistically significant differences were confirmed despite a maximum decrease of 14%, whereas for f_ct, fl._ a significant increase of 11–12% was found.
- The addition of bamboo fibers did not cause statistically significant changes in the ε_y_ strain corresponding to the maximum compressive stress. Relative to the reference series, the value of this strain increased by approximately 11% at a fiber content of 1.0% and by approximately 7% at 1.5%. At the same time, the analysis of the σ_c_–ε curves indicated a similar slope of the quasi-elastic portion and only limited extension of the post-peak response, while the concrete still exhibited a predominantly quasi-brittle failure character.
- The addition of bamboo fibers caused a statistically significant increase in fracture energy under bending. Relative to the reference series, the G_f,δ_ values increased by 20% at a fiber content of 1.0% and by 143% at 1.5%, whereas G_f, CMOD_ increased by 20% and 33%, respectively. At the same time, the DIC analysis showed that in the fiber-reinforced series the principal tensile strain ε_1_ concentration zone at the notch tip was shorter at the moment of maximum load than in the reference series. When considered together with the σ–δ and σ–CMOD curves, this indicates that the increase in fracture energy was primarily associated with delayed microcrack initiation, limited microcrack coalescence, and short-term load retention near the maximum value, without a distinct crack-bridging effect (fiber bridging).
- For the analyzed bamboo fiber variant (1.0–1.5% of cement mass, length of approximately 30 mm, alkali treatment with 2% NaOH for 24 h), the overall effect of the modification on the properties of high-strength concrete was limited and not unequivocally beneficial, and the most pronounced favorable effect of the addition was observed in the fracture-related flexural response of the composite.

## Data Availability

The data that support the findings of this study are available from the corresponding author upon reasonable request.
